# Supplementary material for: The association between variables of cardiopulmonary exercise test and quality of life in patients with chronic Chagas cardiomyopathy (Insights from the PEACH STUDY)
Source: PLoS One. 2022 Dec 15;17(12):e0279086. doi: 10.1371/journal.pone.0279086 (PMC9754173; doi:10.1371/journal.pone.0279086)
Supplement: S1 Table — (DOCX) [file pone.0279086.s001.docx]

Supplementary Table. Study power for adjusted models

| CPET variables | SF-36 Physical functioning domain | |
| --- | --- | --- |
|  | eta-squared | Study power |
| Peak VO_2_ (ml.kg^-1^.min^-1^) | 0.31 | 77% |
| %PPVO_2_ | 0.33 | 81% |
| AT VO_2_ (ml.kg^-1^.min^-1^)^¥^ | 0.50 | 78% |
| Double product (x10^-2^) | 0.15 | 35% |
| O_2_ Pulse (ml/sys) | 0.03 | 9% |
| VE/VCO_2_ slope | 0.06 | 14% |
| OUES (x10^-3^) | 0.09 | 21% |
| HRR ≤ 12 bpm (%) | 0.16 | 38% |
|  | SF-36 Role limitations due to physical problems scale | |
|  | eta-squared | Study power |
| Peak VO_2_ (ml.kg^-1^.min^-1^) | 0.09 | 21% |
| %PPVO_2_ | 0.10 | 23% |
| AT VO_2_ (ml.kg^-1^.min^-1^)^¥^ | 0.17 | 20% |
| Double product (x10^-2^) | 0.10 | 23% |
| O_2_ Pulse (ml/sys) | 0.02 | 8% |
| VE/VCO_2_ slope | 0.02 | 8% |
| OUES (x10^-3^) | 0.07 | 16% |
| HRR ≤ 12 bpm (%) | 0.21 | 51% |
|  | SF-36 Bodily pain scale | |
|  | eta-squared | Study power |
| Peak VO_2_ (ml.kg^-1^.min^-1^) | 0.02 | 8% |
| %PPVO_2_ | 0.03 | 9% |
| AT VO_2_ (ml.kg^-1^.min^-1^)^¥^ | 0.02 | 6% |
| Double product (x10^-2^) | 0.13 | 30% |
| O_2_ Pulse (ml/sys) | 0.01 | 6% |
| VE/VCO_2_ slope | 0.06 | 14% |
| OUES (x10^-3^) | 0.01 | 6% |
| HRR ≤ 12 bpm (%) | 0.18 | 43% |
|  | SF-36 General health perceptions scale | |
|  | eta-squared | Study power |
| Peak VO_2_ (ml.kg^-1^.min^-1^) | 0.07 | 16% |
| %PPVO_2_ | 0.05 | 13% |
| AT VO_2_ (ml.kg^-1^.min^-1^)^¥^ | 0.22 | 27% |
| Double product (x10^-2^) | 0.16 | 38% |
| O_2_ Pulse (ml/sys) | 0.07 | 16% |
| VE/VCO_2_ slope | 0.06 | 14% |
| OUES (x10^-3^) | 0.12 | 27% |
| HRR ≤ 12 bpm (%) | 0.12 | 27% |
|  | SF-36 Physical Component Summary | |
|  | eta-squared | Study power |
| Peak VO_2_ (ml.kg^-1^.min^-1^) | 0.21 | 51% |
| %PPVO_2_ | 0.21 | 51% |
| AT VO_2_ (ml.kg^-1^.min^-1^)^¥^ | 0.36 | 51% |
| Double product (x10^-2^) | 0.20 | 49% |
| O_2_ Pulse (ml/sys) | 0.03 | 9% |
| VE/VCO_2_ slope | 0.02 | 8% |
| OUES (x10^-3^) | 0.11 | 25% |
| HRR ≤ 12 bpm (%) | 0.21 | 51% |
|  | SF-36 Vitality scale | |
|  | eta-squared | Study power |
| Peak VO_2_ (ml.kg^-1^.min^-1^) | 0.09 | 21% |
| %PPVO_2_ | 0.09 | 21% |
| AT VO_2_ (ml.kg^-1^.min^-1^)^¥^ | 0.05 | 8% |
| Double product (x10^-2^) | 0.15 | 35% |
| O_2_ Pulse (ml/sys) | 0.02 | 8% |
| VE/VCO_2_ slope | 0.15 | 35% |
| OUES (x10^-3^) | 0.05 | 13% |
| HRR ≤ 12 bpm (%) | 0.20 | 49% |
|  | SF-36 Social functioning scale | |
|  | eta-squared | Study power |
| Peak VO_2_ (ml.kg^-1^.min^-1^) | 0.02 | 8% |
| %PPVO_2_ | 0.03 | 9% |
| AT VO_2_ (ml.kg^-1^.min^-1^)^¥^ | 0.01 | 6% |
| Double product (x10^-2^) | 0.09 | 21% |
| O_2_ Pulse (ml/sys) | 0.03 | 9% |
| VE/VCO_2_ slope | 0.17 | 40% |
| OUES (x10^-3^) | 0.01 | 6% |
| HRR ≤ 12 bpm (%) | 0.12 | 27% |
|  | SF-36 Role limitations due to emotional problems scale | |
|  | eta-squared | Study power |
| Peak VO_2_ (ml.kg^-1^.min^-1^) | 0.03 | 9% |
| %PPVO_2_ | 0.03 | 9% |
| AT VO_2_ (ml.kg^-1^.min^-1^)^¥^ | 0.00 | 5% |
| Double product (x10^-2^) | 0.13 | 30% |
| O_2_ Pulse (ml/sys) | 0.02 | 8% |
| VE/VCO_2_ slope | 0.16 | 38% |
| OUES (x10^-3^) | 0.04 | 11% |
| HRR ≤ 12 bpm (%) | 0.05 | 13% |
|  | SF-36 Mental health scale | |
|  | eta-squared | Study power |
| Peak VO_2_ (ml.kg^-1^.min^-1^) | 0.01 | 6% |
| %PPVO_2_ | 0.02 | 8% |
| AT VO_2_ (ml.kg^-1^.min^-1^)^¥^ | 0.00 | 5% |
| Double product (x10^-2^) | 0.05 | 13% |
| O_2_ Pulse (ml/sys) | 0.01 | 6% |
| VE/VCO_2_ slope | 0.45 | 97% |
| OUES (x10^-3^) | 0.03 | 9% |
| HRR ≤ 12 bpm (%) | 0.15 | 35% |
|  | SF-36 Mental Component Summary | |
|  | eta-squared | Study power |
| Peak VO_2_ (ml.kg^-1^.min^-1^) | 0.01 | 6% |
| %PPVO_2_ | 0.01 | 6% |
| AT VO_2_ (ml.kg^-1^.min^-1^)^¥^ | 0.01 | 6% |
| Double product (x10^-2^) | 0.09 | 21% |
| O_2_ Pulse (ml/sys) | 0.00 | 5% |
| VE/VCO_2_ slope | 0.31 | 77% |
| OUES (x10^-3^) | 0.03 | 9% |
| HRR ≤ 12 bpm (%) | 0.10 | 23% |

^§^Model adjusted for age, sex, and left ventricular ejection fraction.

^¥^AT VO_2_: n=17.

CPET: cardiopulmonary exercise test; QoL: Quality of Life; SF-36: Medical Outcomes Study 36-Item Short-form of Health Survey; Peak VO_2_: oxygen intake at peak exercise; %PPVO_2_: percent achieved of predicted oxygen uptake at peak exercise; AT VO_2_: oxygen intake at anaerobic threshold; VE/VCO_2_ slope: ventilatory equivalent slope for carbon dioxide output; OUES: oxygen uptake efficiency slope; HRR: first minute heart rate recovery.
