# Supplementary material for: The association between variables of cardiopulmonary exercise test and quality of life in patients with chronic Chagas cardiomyopathy (Insights from the PEACH STUDY)
Source: PLoS One. 2022 Dec 15;17(12):e0279086. doi: 10.1371/journal.pone.0279086 (PMC9754173; doi:10.1371/journal.pone.0279086)
Supplement: Attachment — Submitted filename: Response to Reviewers.docx [file pone.0279086.s002.docx]

**Response to Editor**

**Manuscript ID: PONE-D-22-07420**

**Title: The association between variables of cardiopulmonary exercise test and quality of life in patients with chronic Chagas cardiomyopathy (Insights from the PEACH STUDY)**

Dear editor,

Thank you very much for giving us the opportunity to address the issues raised by reviewers. We also thank the reviewers for their diligent work reviewing the manuscript. We reviewed the manuscript to meet reviewer’s suggestions. Changes were addressed point by point below and marked in yellow in the manuscript. We hope the paper is clearer now and suitable for publication at PLOS ONE. Please, feel free to contact me in case of any addition doubt.

Sincerely yours,

Marcelo Carvalho Vieira, PhD

**Journal requirements:**

When submitting your revision, we need you to address these additional requirements.

1) Please ensure that your manuscript meets PLOS ONE's style requirements, including those for file naming. The PLOS ONE style templates can be found at

https://journals.plos.org/plosone/s/file?id=wjVg/PLOSOne_formatting_sample_main_body.pdf and

https://journals.plos.org/plosone/s/file?id=ba62/PLOSOne_formatting_sample_title_authors_affiliations.pdf".

*Response: Thanks for the notice. We have reviewed the manuscript and ensured that it meets all PLOS ONE style requirements.*

2) In your Data Availability statement, you have not specified where the minimal data set underlying the results described in your manuscript can be found. PLOS defines a study's minimal data set as the underlying data used to reach the conclusions drawn in the manuscript and any additional data required to replicate the reported study findings in their entirety. All PLOS journals require that the minimal data set be made fully available. For more information about our data policy, please see http://journals.plos.org/plosone/s/data-availability.

Upon re-submitting your revised manuscript, please upload your study’s minimal underlying data set as either Supporting Information files or to a stable, public repository and include the relevant URLs, DOIs, or accession numbers within your revised cover letter. For a list of acceptable repositories, please see http://journals.plos.org/plosone/s/data-availability#loc-recommended-repositories. Any potentially identifying patient information must be fully anonymized.

Important: If there are ethical or legal restrictions to sharing your data publicly, please explain these restrictions in detail. Please see our guidelines for more information on what we consider unacceptable restrictions to publicly sharing data: http://journals.plos.org/plosone/s/data-availability#loc-unacceptable-data-access-restrictions. Note that it is not acceptable for the authors to be the sole named individuals responsible for ensuring data access.

We will update your Data Availability statement to reflect the information you provide in your cover letter.

*Response: We apologize for the misunderstanding. The dataset is now available at osf.io/gkavb. Thanks for updating the respective statement.*

3) We note that you have indicated that data from this study are available upon request. PLOS only allows data to be available upon request if there are legal or ethical restrictions on sharing data publicly. For more information on unacceptable data access restrictions, please see http://journals.plos.org/plosone/s/data-availability#loc-unacceptable-data-access-restrictions.

In your revised cover letter, please address the following prompts:

a) If there are ethical or legal restrictions on sharing a de-identified data set, please explain them in detail (e.g., data contain potentially sensitive information, data are owned by a third-party organization, etc.) and who has imposed them (e.g., an ethics committee). Please also provide contact information for a data access committee, ethics committee, or other institutional body to which data requests may be sent.

b) If there are no restrictions, please upload the minimal anonymized data set necessary to replicate your study findings as either Supporting Information files or to a stable, public repository and provide us with the relevant URLs, DOIs, or accession numbers. For a list of acceptable repositories, please see http://journals.plos.org/plosone/s/data-availability#loc-recommended-repositories.

We will update your Data Availability statement on your behalf to reflect the information you provide.

*Response: We apologize for the misunderstanding. The dataset is now available at osf.io/gkavb. Thanks for updating the respective statement.*

Reviewer 1: The present is an interesting paper aiming to evaluate and standardize evaluation of patients with Chagas Disease. Some issues remain to be address.

1) In the abstract some numerical data regarding HR/OR should be added (at least those more relevant)

*Response: We thank the reviewer for the suggestion. We added the β coefficients for all the significant variables in the abstract.*

2) In the introduction it should be better specified the importance of a "standardization" of QoL.

*Response: We appreciated the reviewer suggestion. We added a sentence highlighting the importance of HRQoL standardization.*

3) Was the present analysis pre-specified or not?

*Response: This is a secondary analysis from the PEACH clinical trial using the baseline data. This information was included in the manuscript.*

4) Usually evaluation with time consuming questionnaire is challenging. Who asked the questions to the patients? Do authors recorded a % of complete or not answers?

*Response: The same investigator was responsible for the application of the questionnaires in all patients, which took approximately 20 to 30 minutes. No patient failed to answer the questionnaire. This information was included in the manuscript.*

5) Regarding CPET did authors recorded % of medications?

*Response: The CPET was performed with patients taking all their medications. This information was included in the manuscript. The medications in use by the patients at the time of CPET are described in the Table 1.*

6) Did authors perform a MRI to these patients or not?

*Response: MRI is not a standard exam for evaluation of Chagas cardiomyopathy and was not performed in any patient.*

7) Due to reduced sample size, did authors checked for normality?

*Response: We use generalized linear models with gamma distribution and log-link function that accounts for skewed and heteroscedastic residuals distribution. This information is included in the manuscript.*

8) The rest of the statistical analysis is correct. Usually I do not like when authors correct for "variables known in literature", anyway in this case it is acceptable due to reduced sample size. Please comment

*Response: Many thanks for the comment. Age, sex, and left ventricle ejection fraction are the variables most related to prognosis in Chagas cardiomyopathy and, because of this, they were considered in our model as potential confounders.*

Reviewer 2

1) I suggest the authors put the of the CEP number 2908991, once the CAAE is just a number generated to identify the research project that enters for ethical consideration in the CEP.

*Response: We appreciated the reviewer suggestion. We added the report number after the CAAE.*

2) Consider to mention that this is an arm of a clinical study, since in the clinical trails the main objectives were to evaluate interventions such as exercise, nutritional counseling and pharmaceuticals and not the association of cardiopulmonary variables and quality of life.

*Response: We thank the reviewer for this comment. We rephrased this sentence in the methods section in order to improve clarity.*

3) Was an a priori sample calculation performed? If yes, what was the outcome variable used? If not, I suggest calculating the test power. I understand that as this is an arm of a clinical study, the sample size calculation is not presented and/or applied to the study in question.

*Response: The sample size was calculated only for the clinical trial to detect a clinically significant difference between groups in peak VO_2._ We calculated the study power for the analysis performed in this secondary analysis, which ranged from 5% to 97%. Please see supplementary table. This information was included in the manuscript.*

Reviewer 3: In the present study the authors analyze the correlation between quality of life and exercise performance in patients with Chagas cardiomyopathy. The study is confirmatory of this association in heart failure patients, albeit, as far as I know, not previously evaluated in patients with Chagas cardiomyopathy.

1) The study is well presented, but I suggest the authors to add among the variables VO2 reported as a percent of predicted.

*Response: We thank the reviewer for the suggestion. We include the variable in the analysis as suggested. The results are described in the text, in Tables 1 and 3 and in Figure 1.*

2) The same for the VE/VCO2 relationship slope.

*Response: Mean and standard deviation of the VE/VCO2 slope of participants included in the study were presented in the Table 1. The association between VE/VCO2 slope and QoL scales were presented in the Tables 3 and 4.*

3) The discussion is by far too long and I believe that it should be shortened. Specifically all the parts about autonomic disfunction is mainly speculative and should be deleted.

*Response: We thank the reviewer for this comment. However, although we do agree that the explanations for our findings on autonomic modulation are hypothetical, they are supported by some references cited in the text. We believe that these mechanisms should be presented, even to stimulate further research on the subject.*

Reviewer 4: The study entitled "The association between variables of cardiopulmonary exercise test and quality of life in patients with chronic Chagas cardiomyopathy (Insights from the PEACH STUDY)" is well written and presents an interesting topic of a neglected disease with reflections on other cardiomyopathies. Some aspects need further clarification.

1) Can you provide the interval between CPET and Echocardiography? This is relevant to ascertain that it may reflect precisely its relation to CPET.

*Response: Thank you for the question. CPET and echo were performed within one-week range. This information was included in the manuscript.*

2) On page 8 line 190, you stated that a HRR≤12bpm is probably related to autonomic dysfunction. I suggest considering and presenting data corroborating that. A non-trained individual may present a reduced HRR so looking at a VO2 may help exclude that possibility.

*Response: We thank the reviewer for this comment. It has been demonstrated that a blunted heart rate recovery after exercise testing is a predictor of mortality, both in individuals without cardiovascular disease (Cole et al., 1999) and in individuals with heart failure (Bilsel et al., 2006; Arena et al., 2010). Moreover, Imai et al. (1994) demonstrated that heart rate recovery after exercise testing is blunted in heart failure patients even when compared to age-matched normal control individuals, which presented a statistically significant lower VO_2_. All the above cited references are included in our paper. We made changes to the text to clarify this point.*

3) Was the CPET performed using Beta-blockers? If not, please insert that information. If they were performed using the medications I suggest a paragraph about the possible impact on the results, since nearly all sample was taking them. It may be a study limitation.

*Response: Yes, the CPET was performed with patients taking all their medications, including beta-blockers (93.3%) when applicable. We have included a sentence on study limitations on the impact of beta-blocker use on CPET responses.*

4) Age, gender, and LVEF were your potential confounders, but the literature also presents overweight and obesity as potential confounders (PLoS One. 2021; 16(8): e0255724.). Why did you not consider them? Nearly half of your sample has overweight or obese. Please comment on that.

*Response: We thank the reviewer for the suggestion. Although overweight/obesity has an influence on the CPET response, we chose to include as potential confounders those variables whose impact on the morbidity and mortality of patients with CCC has already been reported in the literature, such as age, gender, and LVEF. In addition, the small sample size prevented us from including more confounding variables in the statistical model.*

5) Although the discussion is comprehensive, I suggest a paragraph on study limitations, discussing beta blockers and overweight impact and the absence of a direct evaluation of the autonomic function, as well as the small sample evaluated.

*Response: Thank you very much for the suggestion. We have expanded the section on study limitations to include the reviewer's suggestions.*
