# Supplementary material for: The association between variables of cardiopulmonary exercise test and quality of life in patients with chronic Chagas cardiomyopathy (Insights from the PEACH STUDY)
Source: PLoS One. 2022 Dec 15;17(12):e0279086. doi: 10.1371/journal.pone.0279086 (PMC9754173; doi:10.1371/journal.pone.0279086)
Supplement: Attachment — Submitted filename: Response to Reviewers R2.docx [file pone.0279086.s003.docx]

To Editorial Board of PLOS ONE

Dear Editor-in-Chief,

**Manuscript ID: PONE-D-22-07420**

**Title: The association between variables of cardiopulmonary exercise test and quality of life in patients with chronic Chagas cardiomyopathy (Insights from the PEACH STUDY)**

Dear editor,

Thank you very much for giving us the opportunity to review our Figure 1. The figure has been revised according to PLOS ONE’s “Figure preparation instructions” and the figure file has been uploaded to the PACE digital diagnostic tool to meet journal requirements. We hope the paper is now suitable for publication at PLOS ONE. Please, feel free to contact me in case of any addition doubt.

Thank you for your consideration.

Sincerely yours,

Marcelo Carvalho Vieira, BPhEd., MSc., PhD

Evandro Chagas National Institute of Infectious Disease, Oswaldo Cruz Foundation, Rio de Janeiro, RJ, Brazil
